# Avian Influenza Virus (H5N1) in Human, Laos

**DOI:** 10.3201/eid1501.080524

**Published:** 2009-01

**Authors:** Pilaipan Puthavathana, Kantima Sangsiriwut, Achareeya Korkusol, Phisanu Pooruk, Prasert Auewarakul, Chakrarat Pittayawanganon, Derek Sutdan, Rungrueng Kitphati, Pathom Sawanpanyalert, Bounlay Phommasack, Khanthong Bounlu, Kumnuan Ungchusak

**Affiliations:** Mahidol University, Bangkok, Thailand (P. Puthavathana, K. Sangsiriwut, A. Korkusol, P. Pooruk, P. Auewarakul); Ministry of Public Health, Nonthaburi, Thailand (C. Pittayawanganon, D. Sutdan, R. Kitphati, P. Sawanpanyalert, K. Ungchusak); Ministry of Health, Vientiane, Laos (B. Phommasack, K. Bounlu)

**Keywords:** Influenza virus (H5N1), human, virus genomic sequence, drug sensitivity, Laos, Thailand, letter

**To the Editor:** The first avian influenza (H5N1) outbreak in poultry in Laos occurred in 2003 and subsided in March 2004 after massive killing of poultry to contain the disease. Extensive surveillance from July 2005 through January 2006 did not detect any influenza virus subtypes in chicken, ducks, quails, and pigs in live bird markets in the Vientiane, Champasak, and Savannakhet Provinces (*1*). Avian influenza virus (H5N1) was reintroduced into Laos in February 2006 but showed a lower incidence. Viruses isolated in this country in 2004 belonged to genotype Z, clade 1, and 2006 isolates belonged to clade 2.3.4 (Appendix Figure, panel A, (*1*).

Avian influenza (H5N1) had not been reported in humans in Laos until February 27, 2007 (*2*). Our patient was a 15-year-old adolescent girl who lived in a suburb of Vientiane where an outbreak of influenza (H5N1) in poultry had been confirmed on February 7, 2007. Influenza-like symptoms developed in the patient on February 10. She was hospitalized in Vientiane with fever and respiratory symptoms on February 15. On February 17, her parents brought her to a private hospital in Nong Khai Province, Thailand. Oseltamivir was prescribed on February 19. On February 20, she was transferred to the Nong Khai Provincial Hospital because of rapid, progressive, severe pneumonia with acute respiratory distress syndrome. When we suspected avian influenza in this patient, clinical specimens were tested.

A diagnosis of infection with avian influenza (H5N1) was based on positive results obtained by reverse transcription–PCR (RT-PCR), viral isolation in MDCK cells inoculated with an endotracheal suction specimen collected on February 22, and a 4-fold increase in neutralizing antibody titers from 80 to 320 in paired blood specimens collected on February 25 and March 1, as assayed against autologous virus. This virus isolate was named A/Laos/Nong Khai 1/07(H5N1). Subsequent samples were collected on February 25 and March 7 (day of death). Results of RT-PCR were positive for the sample collected on February 25 only; virus isolation results were negative for both samples.

The virus was screened for a novel reassorted gene by a multiplex RT-PCR and 8 primer pairs specific for each genomic segment of genotype Z, clade 1 virus (*3*). All segments except the polymerase A (PA) segment were amplified, which indicated that the new virus was different from genotype Z viruses. The viral genome was sequenced and submitted to GenBank (accession nos. EU499372–EU499379 for hemagglutinin, nonstructural protein, matrix protein, nucleoprotein, PB1, PB2, neuraminidase, and PA genes, respectively). Phylogenetic analysis showed that this virus belonged to genotype V (Appendix Figure, panel B) (*4*); phylogenetic analysis of the hemagglutinin gene (www.who.int/csr/disease/avian_influenza/smaltree.pdf) showed that it belonged to clade 2.3.4 (Appendix Figure, panel A).

Protein sequence at the hemagglutinin cleavage site harbored many basic amino acids (RERR_RKR). One amino acid deletion and 1 amino acid change were found when compared with RERRRKKR, which is present in most avian influenza viruses (H5N1). There was no change in receptor binding site. This virus had glutamic acid at aa 627 in the PB2 protein, aspartic acid at aa position 92 in nonstructural protein 1, and 5 aa deletions at positions 80–84 in the nonstructural protein 1. Analysis of the neuraminidase gene showed a 20-aa deletion in the stalk protein; there was no mutation of histidine to tyrosine at aa position 274, a position shown to be the oseltamivir resistance marker in the neuraminidase 1 viral genome (*5*). Mutations in the matrix 2 gene showed that amantadine resistance was not present in our virus (*6*). Our in vitro assay (*7*) showed that this virus was sensitive to oseltamivir and amantadine.

Since 2003, genotype V influenza viruses (H5N1) have been reported in some East Asian countries. Genetic diversity in the hemagglutinin gene has classified those genotype V viruses into distinct clades. Viruses from avian species in South Korea in 2003 and Japan in 2004 (*8*,*9*) belong to clade 2.5. A/chicken/Shanxi/2/2006 isolate belonged to clade 7. Human cases in People’s Republic of China, i.e., A/China/GD01/06, A/Shenzhen/406H/06, A/Jiangsu/1/2007, and A/Jiangsu/2/2007, belong to clade 2.3.4, the same clade as A/chicken/Thailand/NP-172/2006 and the virus from our study.

Highly pathogenic avian influenza viruses (H5N1) that caused outbreaks in Thailand since 2004 belong to genotype Z, clade 1. Introduction of genotype V clade 2.3.4 virus, A/chicken/Thailand/NP-172/2006, to Nakhon Phanom Province occurred in November 2006 (*10*), the same year that clade 2.3.4 virus was introduced into Laos (Appendix Figure, panel A). On the basis of hemagglutinin gene phylogeny, A/Laos/Nong Khai 1/2007 is closely related to A/chicken/Nong Khai/NIAH 400802/2007 and A/chicken/Thailand/NP-172/2006. Phylogenetic analysis suggested that viruses from these 2 countries shared the same origin. There was extensive movement across the Mekong River even before the bridge linking Nong Khai from Vientiane was opened. However, the route of transmission of genotype V viruses from east Asian to Southeast Asian countries could not be elucidated.

## Supplementary Material

Appendix Figure Phylogenetic analysis of avian influenza viruses (H5N1). A) hemagglutinin genes and B) polymerase A genes. Pseudosampling = 1,000. Known genotype V viruses are indicated in red, and genotype Z viruses are indicated in blue. Numbers on the right in braces indicate clades and subclades. Scale bars indicate genetic distances between sequences of different taxa. HK, Hong Kong.
