# Supplementary figures and images for: Avian Influenza Virus (H5N1) in Human, Laos
Source: Emerg Infect Dis. 2009 Jan;15(1):127–9. doi: 10.3201/eid1501.080524 (PMC2660695; doi:10.3201/eid1501.080524)

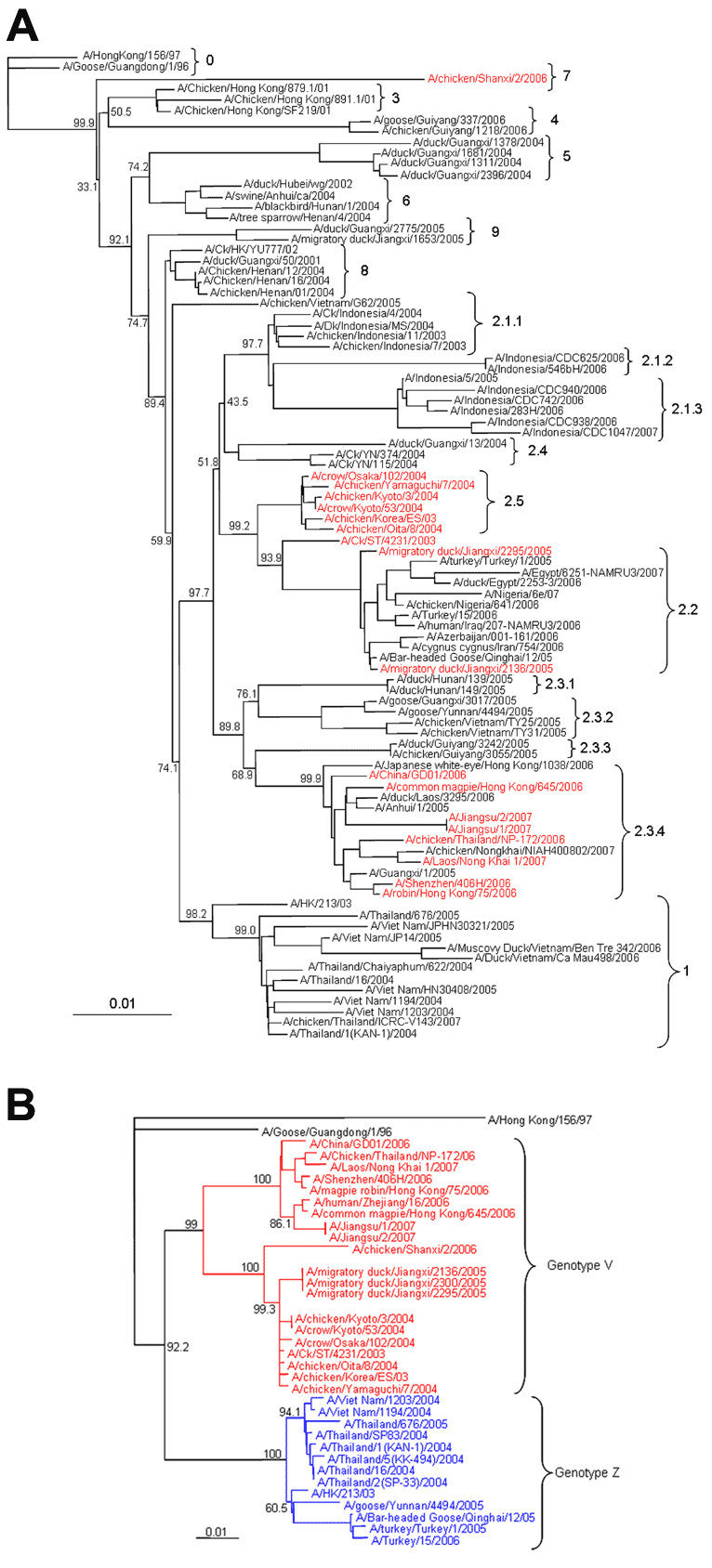

Supplement: Appendix Figure — Phylogenetic analysis of avian influenza viruses (H5N1). A) hemagglutinin genes and B) polymerase A genes. Pseudosampling = 1,000. Known genotype V viruses are indicated in red, and genotype Z viruses are indicated in blue. Numbers on the right in braces indicate clades and subclades. Scale bars indicate genetic distances between sequences of different taxa. HK, Hong Kong. [file 08-0524_app-s1.gif]
